# Assessment of inducible clindamycin resistance and Hyper Variable Region (HVR) of *mec*A gene in clinical staphylococci

**DOI:** 10.12669/pjms.36.2.665

**Published:** 2020

**Authors:** Amir Afzal Khan, Jahanzaib Farooq, Madiha Abid, Rabaab Zahra

**Affiliations:** 1 Amir Afzal Khan, Department of Microbiology, Quaid-i-Azam University, Islamabad, Pakistan; 2 Jahanzaib Farooq, Department of Microbiology, Quaid-i-Azam University, Islamabad, Pakistan; 3 Madiha Abid, Department of Microbiology, Quaid-i-Azam University, Islamabad, Pakistan; 4 Rabaab Zahra, Department of Microbiology, Quaid-i-Azam University, Islamabad, Pakistan

**Keywords:** Clindamycin inducible resistance, D-test, HVR region, *mec*A, Staphylococci

## Abstract

**Objective:**

To study the prevalence of inducible clindamycin along with vancomycin and methicillin resistance and assessment of hyper variable region (HVR) of *mec*A gene among different clinical isolates of *Staphylococcus* spp.

**Methods:**

A total of 176 clinical isolates of *Staphylococci* were collected from Pakistan Institute of Medical Sciences (PIMS), Islamabad during 2014-2015. The sample sources were pus, blood, urine, sputum, tracheal secretions and tissue fluids. Bacterial identification was done by colony morphology and biochemical tests. Kirby-Bauer disc-diffusion method was carried out to assess the susceptibility against different antibiotics. Minimal inhibitory concentrations (MICs) were done for vancomycin resistance. Double Disk Diffusion test (D-test) was used to detect the clindamycin inducible resistance. PCR was performed to detect *erm*(C), *mec*A and HVR genes.

**Results:**

Clindamycin inducible resistance among Staphylococcal isolates was found to be 7%, whereas in *S. aureus* it was 4%, and in coagulase negative *Staphylococci* (CoNS) it was 11%. The highest resistance was observed against fosfomycin, fusidic acid and cefoxitin. Vancomycin resistance was observed in 23 isolates (13%) of Staphylococci. *erm*(C), *mec*A and HVR genes were found in 18%, 50% and 42% respectively.

**Conclusions:**

D-test must be performed routinely to avoid clindamycin failure. A high level of resistance against vancomycin in Staphylococcal isolates is a concern for public health.

## INTRODUCTION

*S. aureus* and Coagulase-negative *Staphylococci* (CoNS) are considered to be one of the leading causes of hospital and community acquired infections. Resistance to methicillin and vancomycin in *Staphylococci* is well-known and it is important to find alternatives that can be used in case of methicillin resistant *S. aureus* (MRSA) and vancomycin resistant *S. aureus* (VRSA). MRSA strains carry mobile genetic elements, recognized as Staphylococcal cassette chromosome *mec* (SCC*mec*) that has a number of genes where the most important is *mec*A and genes that regulate the expression of *mec*A.^1^ *mec*A gene is responsible for resistance to methicillin and other β-lactam drugs in *S. aureus* and encodes a 78-kDa penicillin-binding protein 2a (PBP2a).^2^

In 1991, a new region between IS431-*mec* and the *mec*A gene in SCC*mec* was described;^3^ which was named as the hyper variable region (HVR) due to DNA length polymorphism. One MRSA strain’s HVR region was sequenced and ten repeat units of 40bp each were identified.^3^ Most of the studies either have analyzed the hybridization patterns of these direct repeat units PCR products or compared the gel band sizes^4^ while direct repeat unit (dru) region was sequenced by one study.^5^ Since the number of these repeat units may be different among isolates, the amplification of HVR region can be used to type and classify MRSA strains.

The macrolide-lincosamide-streptogramin B (MLSB) is a group of antibiotics used to treat different infections caused by Staphylococci.^6^ Clinicians avoid clindamycin administration, when erythromycin resistance is present, as erythromycin can induce clindamycin resistance in clindamycin sensitive strains by activating *erm*(C) gene. *erm*(C) is usually located on small plasmids and is responsible for inducible or constitutive resistance to erythromycin.^7^ This study was designed to analyze the prevalence of clindamycin inducible resistance along with vancomycin and methicillin resistance and assessment of hyper variable region in MRSA, among different clinical isolates of *Staphylococci* spp.

## METHODS

### Bacterial isolates

A total of 176 clinical isolates of *Staphylococci* spp. were collected from Pakistan Institute of Medical Sciences (PIMS), Islamabad during 2014-2015. Out of these isolates, 138 (78%) were from out-patients and 38 (22%) were from in-patients. Main sample sources were pus followed by blood, urine, tracheal secretions, sputum and tissue fluids.Ethical Approval (No. F.1-1/2015/ERB/SZABMU/ dated May 20, 2016) was obtained from Ethics Review Board of Shaheed Zulfiqar Ali Bhutto Medical University, PIMS, Islamabad.

### Bacterial identification

Identification was done by colony morphology on mannitol salt agar and biochemical tests that included catalase, slide and tube coagulase, and DNase tests.

### Antibiotic Susceptibility Testing

Antibiotic susceptibility was done by Kirby-Bauer disk diffusion method^8^ against a panel of antibiotics which included cefoxitin (30µg), linezolid (30µg), rifampin (5µg), fusidic acid (10µg), fosfomycin (50µg), tigecycline (15µg), tetracycline (30µg), chloramphenicol (30µg), ciprofloxacin (5µg), gentamicin (10µg), clindamycin (2µg), erythromycin (15µg), and sulfamethoxazole/trimethoprim (25µg) (Oxoid, UK). *S. aureus* ATCC 25923 was used as quality control strain. Results were interpreted according to the Clinical and Laboratory Standard Institute (CLSI) guidelines 2015. Minimum inhibitory concentrations (MICs) were performed for vancomycin (Sigma-Aldrich) using agar dilution method.^9^

### Double Disk Diffusion test (D-test)

D-test was performed to detect the inducible clindamycin resistance. Bacterial lawn was prepared on Mueller Hinton agar and antibiotic discs of clindamycin (2µg), and erythromycin (15µg) were placed 15-20 mm apart. Plates were incubated at 37^°^C overnight. Three phenotypes were interpreted according to CLSI guidelines 2014. *Macrolide Type-B streptogramin (MSB) phenotype*: resistance to erythromycin (zone size ≤ 13mm) and sensitivity to clindamycin (zone size ≥ 23mm) with a circular zone of inhibition around clindamycin (D-test negative). *Inducible macrolides-lincosamides-streptogramin B (iMLSB) phenotype:* resistance to erythromycin (zone size ≤ 13mm) and sensitive to clindamycin (zone size ≥ 23mm) with D-shaped zone of inhibition around clindamycin (D-test positive). *Constitutive macrolides-lincosamides-streptogramin B (cMLSB) phenotype:* resistance to both erythromycin (zone size ≤ 13mm) and clindamycin (zone size ≤ 14mm) with circular zone of inhibition following CLSI guidelines 2015.

### Molecular detection of clindamycin inducible resistance erm(C), mecA and HVR genes

Genomic DNA was isolated using lysis method previously described.^10^ Phenotypically D-test positive isolates were screened for the presence of *erm*(C) gene while *mec*A and *HVR* genes were screened in MRSA isolates by PCR using already published primers.^11^-^13^HYPERLINK \l “_ENREF_12” \o “Senna, 2002 #21” PCR conditions were as following; initial denaturation at 95°C for 5 minutes, 35 cycles of 95°C for one minute, annealing at 47°C for one minute for *erm*(C), 59°C for *mec*A and 58°C for *HVR*, extension at 72°C for 1 minute followed by the final extension at 72°C for 10 minutes. PCR products were run on 2% agarose gel and visualized in UV transilluminator (UVItec, EEC).

### Statistical Analysis

Statistical analysis was carried out to find association between resistance profiles of organisms using GraphPad Prism software version 7.04. A *p-*value less than 0.05 was considered statistically significant.

## RESULTS

Out of a total of 176 Staphylococcal isolates 51% (n=90) were identified as *S. aureus* of which 69% (n=62) were MRSA and 31% (n=28) were methicillin sensitive *S. aureus* (MSSA). A total of 49% (n=86) isolates were identified as CoNS, in which 93% (n=80) were methicillin resistant coagulase negative *Staphylococci* (MRCoNS) and 7% (n=6) were methicillin sensitive coagulase negative *Staphylococci* (MSCoNS). Major sample sources were pus (48%), followed by blood (29%), tracheal secretions (8%), urine (4%), sputum (3%), tip of drain (3%), tissue fluids (2%), catheter tip (1.3%), nasal swabs (1%) and semen (0.7%).

### Antibiotic Resistance Profile and Minimal inhibitory concentrations (MICs)

Among all *S. aureus* strains, maximum resistance was observed against fosfomycin followed by fusidic acid, cefoxitin, tetracycline and ciprofloxacin. Least resistance was observed against chloramphenicol, gentamicin and rifampicin. Among CoNS, the maximum resistance was observed against fosfomycin and cefoxitin followed by linezolid, gentamicin, fusidic acid and tetracycline. The least resistance was observed against chloramphenicol and tigecycline. We found a significant correlation of antibiotic resistance with CoNS as compared to *S. aureus* (*p*=0.001). The detailed resistance profile of all isolates and statistical comparison are shown in Table-I. From a total of 176, 23 isolates of *Staphylococci* showed resistance to vancomycin using Kirby Bauer method so MICs were performed on these 23 resistant isolates using agar dilution method, which showed that 6 (7%) isolates were VRSA having MICs more than 16 µg/mL and 17 (20%) were vancomycin resistant coagulase negative *Staphylococci* (VRCoNS). Out of 17 isolates of VRCoNS, one isolate showed intermediate resistance with MIC of 8 µg/mL and 16 isolates had MICs greater than 32 µg/mL.

**Table-I:** Resistance profile of all Staphylococcal isolates.

Antibiotics	Resistant profile of S. aureus (%)	Resistant profile of CoNS (%)	p-value
Linezolid (LZD)	37	90	0.0001
Tigecycline (TGC)	39	33	0.372
Tetracycline (TE)	51	66	0.0301
Ciprofloxacin (CIP)	50	59	0.1739
Rifampin (RD)	28	43	0.0174
Fosfomycin (FOS)	90	95	0.1795
Fusidic Acid (FD)	87	81	0.3408
Clindamycin (DA)	37	52	0.0227
Erythromycin (ER)	42	66	0.0007
Cefoxitin (FOX)	71	94	0.0001
Gentamicin (CN)	28	83	0.0001
Chloramphenicol (C)	4	30	0.0001
Sulfamethoxazole (SXT)	49	58	0.1566

### Phenotypes of Staphylococci on the basis of D-test

**Fig.1:**
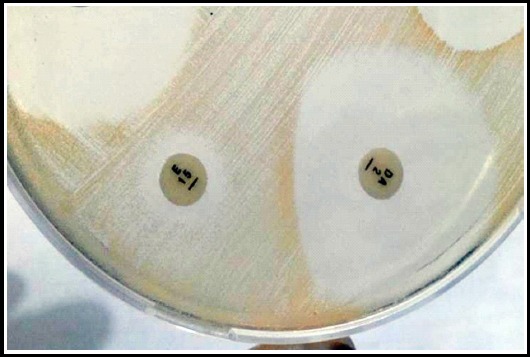
A representative Mueller Hinton agar plate showing positive D-test which is a D type zone around clindamycin (DA) disc.

Out of a total of 176 staphylococcal isolates, 152 isolates were tested for inducible clindamycin resistance using D-test (Fig.1). Out of 152 isolates, MSB phenotype was observed in 27 (18%) isolates, cMLSB phenotype was observed in 114 isolates (75%) while 11 (7%) isolates showed iMLSB phenotype. Among these 11 isolates, three isolates were MRSA, one was MSSA and seven were MRCoNS. Overall, inducible clindamycin resistance in *S. aureus* was 4% and among CoNS it was 11%.

### Molecular detection of HVR, erm(C) and mecA gene

**Fig.2:**
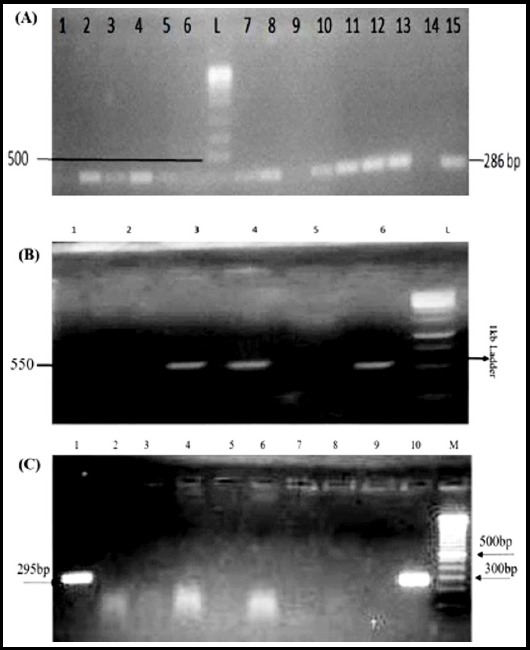
Representative gel images showing PCR products of *mec*A, HVR and *erm*(C) genes. **(A)** Lane L shows ladder of 1kb, Lanes 2-8, 10-13, and 15 show shows amplification of *mec*A gene (286bp) and Lane-1 shows negative control. **(B)** Lane L shows ladder of 1 kb, Lanes 3, 4 and 6 show amplification of HVR gene (550 bp) Lanes 1, 2 and 5 show negative results. **(C)** Lane M shows 100 bp ladder, Lanes 1 and 10 show amplification of *erm*(C) gene (295bp), Lanes 2-9 show negative results.

Out of 11 isolates that showed iMLSB phenotype, only two (18%) isolates (D-test positive) showed the presence of *erm*(C) gene (Fig.2C) and these were MRSA. Out of 62 MRSA, 50% were positive for *mec*A gene (Fig.2A) and 48% isolates were found negative for *mec*A gene. We also screened MSSA for *mec*A gene and all were found to be negative. Out of 31 *mec*A positive isolates, 68% were from male patients while 32% were from female patients. Out of MRSA isolates 26 (42%) were found to be positive for HVR (Fig.2B) whereas 36 (58%) isolates did not reveal the presence of HVR.

## DISCUSSION

The prevalence of MRSA in this study was reported as 69% which is higher than previous reports from Pakistan where a range of 5% in 1989 and up to 52% in 2017 have been reported.^14^,^15^ This indicates a continuous increase in the circulation of this organism in clinical settings. Prevalence of methicillin resistance among CoNS in this study was 93% which is higher than previous reports of 70% oxacillin resistance in CoNS.^16^ The prevalence of inducible clindamycin resistance among all *Staphylococci* was observed to be 7% while among *S. aureus* isolates it was 4%. All of the CoNS were methicillin resistant. Clindamycin inducible resistance is difficult to be noticed in routine antibiotic susceptibility testing if clindamycin and erythromycin discs are not placed adjacent to each other. Clinicians end up prescribing clindamycin without knowledge of whether the particular strains of *Staphylococci* are positive for clindamycin inducible resistance or not. D-test is used to detect inducible resistance in which both antibiotic discs are placed adjacent to each other with the distance of 15-20mm. Inducible resistance is one of the factors of clindamycin therapeutic failure. So D-test must be performed in routine diagnostic laboratories for analysis of clinical Staphylococci.

One study from Peshawar has reported 16% inducible clindamycin resistance in MRSA isolates^17^ while a study from Karachi reported 72% inducible clindamycin resistance phenotype in *S. aureus* isolates.^18^ Prevalence of clindamycin inducible resistance in CoNS in this study was 11%. There is no data available on prevalence of clindamycin inducible resistance among CoNS from Pakistan while a study from India reported 7.56% inducible clindamycin resistance in CoNS and 16.4% in *S. aureus*.^19^ This shows that prevalence of clindamycin inducible resistance varies from region to region. In the present study, *erm*(C) gene was detected in only two isolates showing phenotypic inducible clindamycin resistance. In one study from Iran, three *S. aureus* isolates with positive D-test were negative for *erm* genes by PCR.^20^ Similar findings have been reported where 17%^21^ and 33%^22^ of *S. aureus* strains were negative for *erm*(A) and *erm*(C) genes. A positive D-test suggests the presence of an *erm* gene that could result in inducible clindamycin resistance and clinical failure. Absence of *erm* genes in positive D-test isolates suggests the role of other factors or genes.

In the current study, prevalence of *mec*A gene was reported as 50% which is slightly higher from the report of 48.1% from Peshawar.^17^ MRSA isolates subjected to HVR-PCR generated amplification products that ranged from 290bp to 650bp in size with 3 HVR types: HVR 2 (400bp), HVR 5 (550bp), HVR 6 (600bp). One study reported eight types of HVR which were HVR types 3, 5, 7, 8, 9, 10, 11, and 12 direct repeat units (DRUs).^12^ Another study from Iran reported HVR types 1-11.^23^ The differences in the HVR region could be due to the geographical variation, different samples size, and host genetic factor(s).

In the current study, vancomycin resistance in MRSA was recorded as 7% while in CoNS it was 20%. One study from Pakistan recorded high levels of vancomycin resistance in MRSA as 13% using E-test method.^24^ Reason for increase in rate of resistance to vancomycin could be inadequate use of vancomycin and transfer of vancomycin resistance genes (*van*A-C) from other vancomycin resistant organisms such as *Enterococcus*. Particularly our clinicians should be concerned about these reports on vancomycin resistance so that they can prescribe vancomycin accordingly to avoid therapeutic failure. Resistance to tigecycline against *S. aureus* in the present study was observed to be 39% which is a concern as previous reports from Pakistan have reported 100% susceptibility to tigecycline.^15^,^25^ In the current study, resistance to linezolid was reported as 37% in *S. aureus* isolates while in CoNS it was 90%. Previous study conducted in Pakistan reported *cfr* gene in 78% of linezolid resistant *S. aureus* isolates^24^ which is higher than our results, although we observed high resistance of linezolid in CoNS. The increase in rate of resistance against linezolid is very alarming, particularly among CoNS which are developing resistance to other antibiotics as well.

## CONCLUSION

This is the first report of clindamycin inducible resistance in coagulase negative *Staphylococci* from local strains in Islamabad. Increasing levels of resistance in coagulase negative *Staphylococci* is a point of concern for public health and the prescription of antibiotics in these cases should be given with caution. We also suggest the use of D-test in routine antibiotic susceptibility analysis in order to cater the inducible clindamycin resistance.

### Author`s Contribution:

**AAK**: Performed the experiments and prepared draft of paper.

**JF**: Performed the experiments, analyzed data and helped in writing the manuscript draft.

**MA**: Performed experiments, analyzed data.

**RZ**: Conception and design of research, analysis interpretation of the data, approval of final version, is responsible for integrity of research.
